# Minimally invasive versus open chevron osteotomy for hallux valgus correction: a randomized controlled trial

**DOI:** 10.1007/s00264-018-4006-8

**Published:** 2018-06-04

**Authors:** Gerhard Kaufmann, Dietmar Dammerer, Felix Heyenbrock, Matthias Braito, Lorenz Moertlbauer, Michael Liebensteiner

**Affiliations:** 1 Orthopaedic and Foot Center Innsbruck (OFZ Innsbruck), Innrain 2, 6020 Innsbruck, Austria; 2 Orthopaedic Department of the Medical University of Innsbruck, Anichstrasse 35, 6020 Innsbruck, Austria; 3 Department of Orthopaedics, Medical University of Innsbruck, Anichstraße 35, 6020 Innsbruck, Austria

**Keywords:** Bunion, Chevron, Hallux valgus, Minimally invasive, Percutaneous

## Abstract

**Purpose:**

The purpose of this study was to compare a minimally invasive chevron osteotomy technique (MIS group) and the well-established open chevron technique (OC group) for correction of hallux valgus deformity.

**Methods:**

Patients who were scheduled to undergo a hallux valgus surgery by means of a distal chevron osteotomy were randomly assigned to one of the two groups. Pre-operatively, six weeks, 12 weeks, and nine months post-operatively the following outcome parameters were determined: Visual Analog Scores (VAS) of pain, the American Orthopedic Foot and Ankle Society (AOFAS) forefoot score, radiographic outcome measures, range of motion (ROM), and patient satisfaction.

**Results:**

Forty-seven cases were analyzed (25 MIS group; 22 OC group). Both operative techniques achieved significant correction of the hallux deformity. The intermetatarsal angle (IMA) improved from 15.1° to 5.8° in the OC and from 14° to 6.8°in the MIS group, whereas the hallux valgus angle (HVA) improved from 28.3° to 8.5° in the OC versus 26.4° to 6.9° in the MIS group. No significant differences were observed between the groups by any of the determined outcome parameters. Regarding patient satisfaction, statistically significant differences were found between MIS and open surgery 12 weeks post-operatively in favour of the MIS group (*p* = 0.022).

**Conclusion:**

With the minimally invasive chevron osteotomy, radiological and clinical outcome is comparable to the open technique.

## Introduction

Multiple different surgical techniques have been established for hallux valgus surgery so far, with each technique having its unique advantages and limitations. The distal chevron method was first described in 1981 [1] and is widely accepted as a method for correcting mild to moderate hallux valgus deformities [2]. Numerous publications presenting the radiological outcome of this surgical technique [3, 4] and the clinical outcome by means of well-established score systems [5, 6] have been published and make this technique, representing distal procedures, today’s benchmark.

As a consequence of the open approach to the joint, scarring and decreased range of motion can occur [7]. Patients’ demands and soft tissue scarification with open surgery have caused several minimally invasive techniques to be published in the last few years [8–11]. Given to the current literature, these minimally invasive techniques claim, as one of their advantages compared to open techniques, to have minor soft tissue damage, reduced surgical time, and faster recovery, while same stability of correction and clinical outcome [8–11]. Therefore it seems that minimally invasive techniques are on the rise but the efficiency and stability of correction, as well as the clinical outcome of these minimally invasive techniques, have been discussed controversially. Whereas most studies presented good clinical and radiological results [10, 12], the group Romero et al. found insufficient radiographic HVA correction. However they stated low post-operative pain levels and high patient satisfaction [13]. The study by Lee was prospective, but compared the minimally invasive chevron and the scarf technique [10], which is a distinct issue. In a systematic review of minimally invasive hallux surgery, a clear recommendation could not be made although early results of multiple studies are encouraging [14]. To date and due to the previously published literature, prospective-comparative studies comparing open versus MIS chevron techniques have not been published and the difference in any of the outcome parameters between the two surgical procedures (open vs. minimal invasive) is not clear given in the current literature.

Given the above-mentioned lack of evidence, it was the aim of the study to prospectively compare conventional open chevron osteotomy and MIS chevron osteotomy. It was hypothesized that the two techniques would show significant differences with regard to clinical score outcome (hypothesis 1), radiographic outcome (hypothesis 2), range of motion (ROM) of the first metatarsophalangeal joint (hypothesis 3), and patient satisfaction (hypothesis 4).

## Material and methods

### Study design

Patients on the waiting list for a distal chevron osteotomy for hallux valgus were considered for participation. Exclusion criteria were (1) age < 16 years; (2) previous first metatarsal osteotomy or soft tissue intervention for hallux valgus deformity; (3) instability of the first tarsometatarsal joint, defined as abnormal painful motion in this joint; (4) osteoarthritis of the first metatarsophalangeal joint, (5) preoperative hallux valgus angle (HVA) of less than 20° [15, 16]; or (6) an intermetatarsal angle (IMA) of less than 10° [17].

Eligible patients were then invited to participate and provided written informed consent. All patients failed conservative treatment. Patients were randomized to either the OC group or the MIS group. Assignment to these groups was done by lot. After calculation of sample size, we gathered the same amount of envelopes as feet had to be included to the study in a black box. One half was marked with the letters “OC,” the other with “MIS.” The day before surgery, for every patient, an envelope was drawn and administered to the surgical group marked on the envelope. Since several patients were treated bilaterally, the cohort is of different sizes.

### Surgical technique

In the OC group, a 4-cm-long dorsomedial skin incision was made to perform a V-shaped osteotomy. The apex of the osteotomy was centered 1–2 mm superior to the centre of the metatarsal head, the angle of the chevron was 60° to 90°. To avoid shortening, the osteotomy was directed toward the centre of the third metatarsal head with a slight plantarization of 5° to 10°. The metatarsal head was shifted laterally, sesamoid position was controlled by sight. Fixation was achieved by one cannulated screw (3.0 or 2.5 mm—FRS—Screw [Fusion and Reconstruction System], DePuy-Synthes, Saint Priest, France). Prominent bone ridges were resected with a saw. The distal soft tissue procedure was performed through the same dorsomedial incision. The adductor hallucis tendon was detached from its insertion at the phalangeal bone and from the lateral border of the fibular sesamoid. The transverse intermetatarsal ligament was released and a T-shaped capsulotomy was performed to allow reposition of the sesamoids. Closing of the medial capsula was performed with sutures of coated number 1 polyglactin 910 (Vicryl, Ethicon, Johnson & Johnson). The skin was closed with nylon sutures 4–0.

**Fig. 1:**
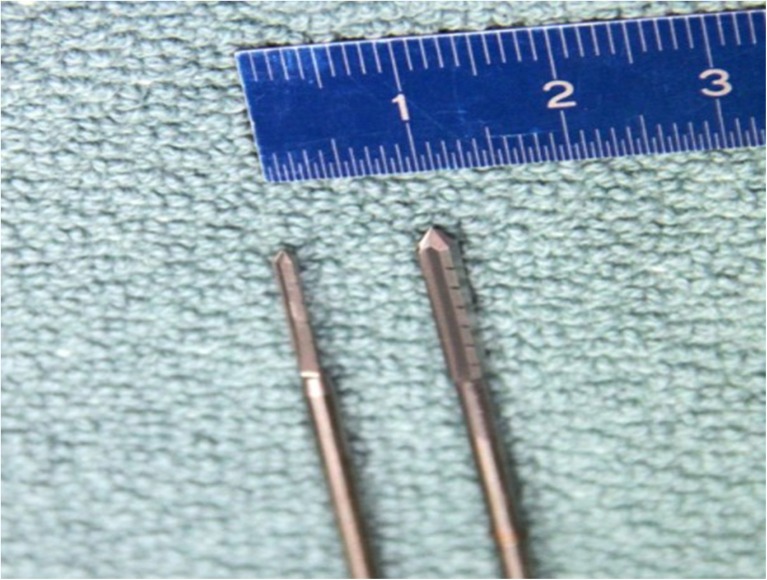
Showing both reamers (2.1 mm on the left, 3.1 mm on the right side)

**Fig. 2:**
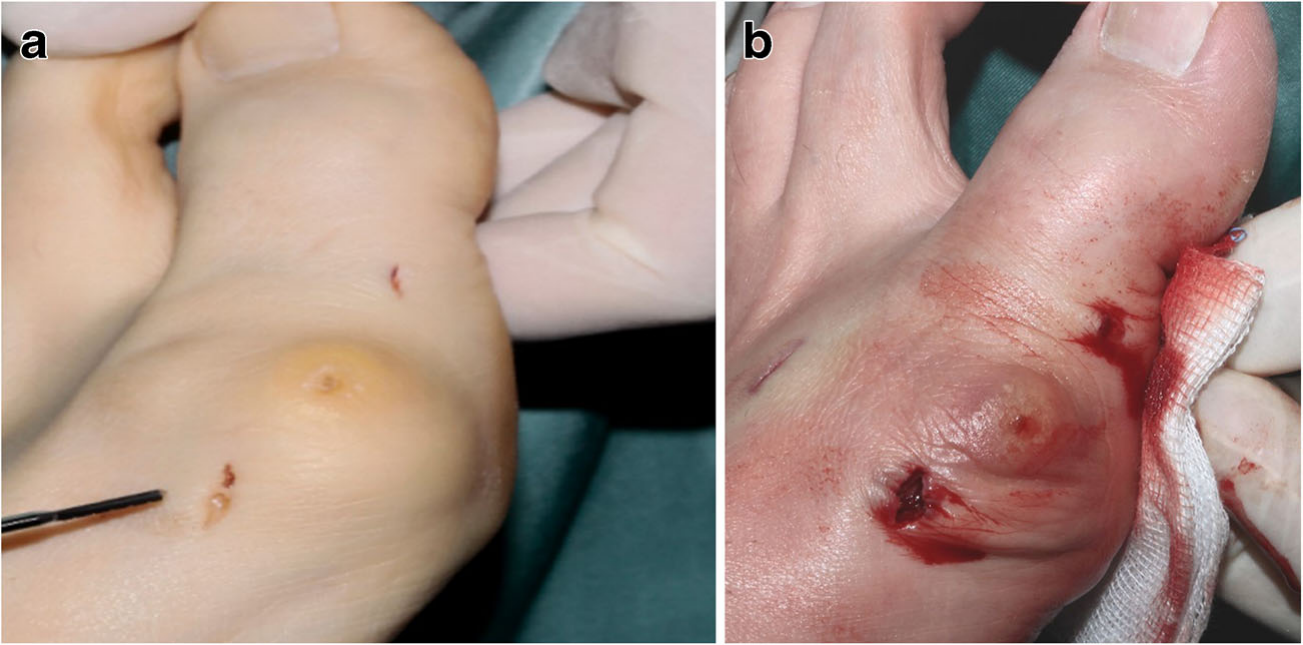
Showing the incisions before (**a**) and after opening the hydraulic blood barrier (**b**) in a case with combination of chevron and akin osteotomy

**Fig. 3:**
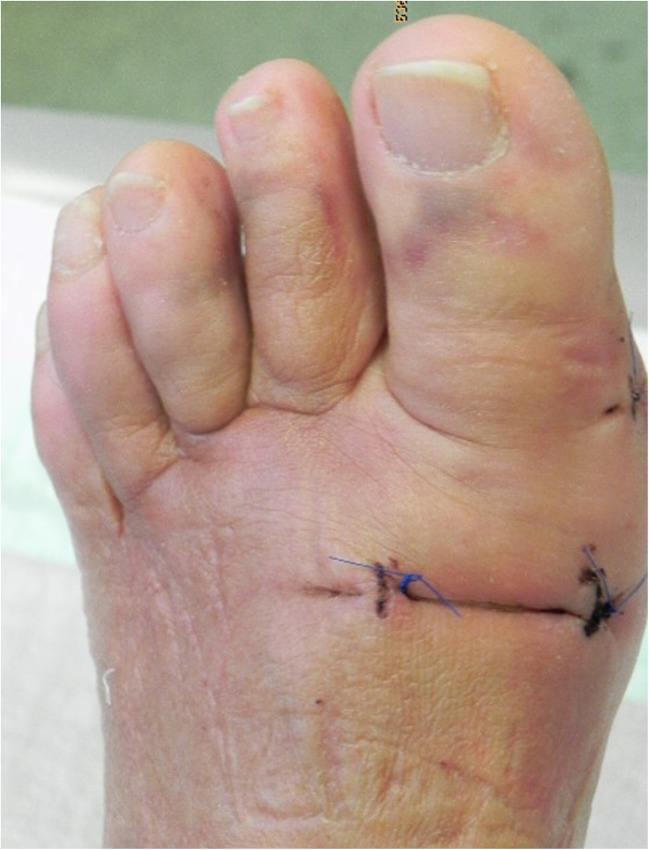
Skin sutures on day 8 after surgery (chevron and akin osteotomy)

In the MIS group, the osteotomy was performed percutaneously through a dorsomedial incision of 3–5 mm. An electric motor-driven machine with variable speed was used for the osteotomy (NSK Prado Surgic Pro non-optic, NSK, IL, USA). Speed was limited to 8000 rpm. To prevent overheating, the reamer was frequently rinsed with sterile saline. The medial eminence was excised with a straight reamer of 2.1 or 3.1 mm (Newdeal SAS, Saint Priest, France). With the 2.1-mm reamer, the V-shaped osteotomy was performed (Fig. 1). The apex of the osteotomy was identified by fluoroscopy and centered 1–2 mm superior to the centre of the metatarsal head. The tip of the reamer was directed toward the centre of the head of the third metatarsal with a plantarisation of 5° to 10°. After reaming, bone debris was washed out with sterile saline. A lateral soft tissue release was undertaken through a separate lateral incision of 3–5 mm as well (Fig. 2 A + B). The distal fragment was shifted laterally with a slight varus rotation to re-align the joint line. Fixation was achieved with a 1.2-mm K wire that was inserted through the same incision as used for the osteotomy. The K wire was bent at the bone insertion point and cut with an overhang of 3–5 mm. Residual bone ridges were reamed and bone debris washed out. Position of the metatarsal head and the K wire was controlled by fluoroscopy. The skin was closed with a nylon suture 4–0 (Fig. 3).

Post-operative treatment was standardized over both groups. Soft dressings were applied and patients were allowed to weight bear immediately in a custom-made hallux valgus shoe (Ofa GmbH, Bamberg, Germany). After suture removal at two weeks, post-operative wound cover was reduced to allow full movement. The surgical shoe was discarded at six weeks.

### Outcome parameters

**Fig. 4:**
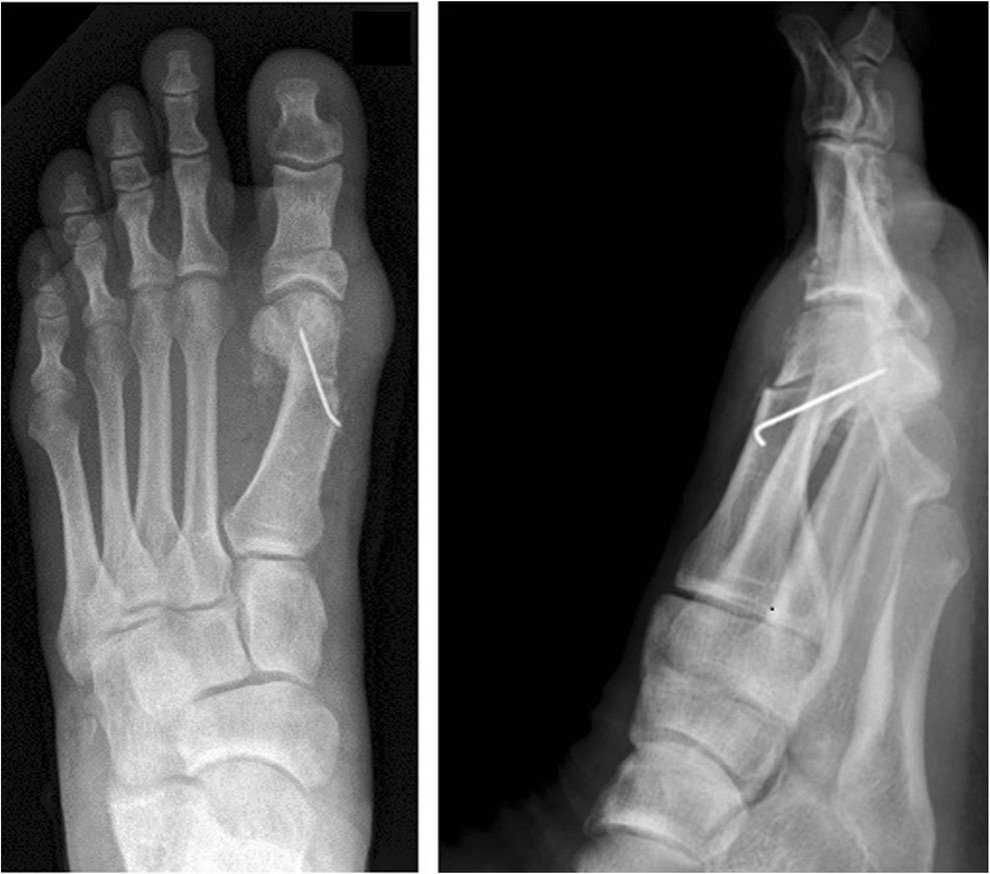
Standing anteroposteriorradiograph (left foot) showing result after a minimally invasive chevron and akin osteotomy

All outcome parameters were determined pre-operatively and six weeks, 12 weeks, and nine months post-operatively. We did not lose patients for follow-up. Weight-bearing radiographs (Fig. 4) were analyzed in digital manner using the Icoview software (syngo.share, ITH icoserve healthcare GmbH, Siemens, Innsbruck, Austria). The radiographic outcome measures used in this study were as follows: the HVA, which is the angle between the midshaft axes of the first metatarsal bone and the proximal phalangeal bone [18]; the intermetatarsal angle (IMA) which is the angle between the midshaft longitudinal axis of the first and second metatarsal bone. In the post-operative radiographs, it was measured as the angle subtended by the lines from center head to centre base of the first and second metatarsal [18–20].

Clinical outcome was measured in terms of passive range of motion (ROM) of the first metatarsophalangeal joint. Evaluation was performed using a goniometer in maximal plantarflexion as well as maximal dorsiflexion. ROM outcome was categorized in three groups (R1 (ROM < 30°); R2 (ROM 31°–75°); R3 (ROM > 75°); pain was assessed using the Visual Analog Scale (VAS 10). In addition, the outcome was rated with the AOFAS forefoot score (6) (American Orthopedic Foot & Ankle Society Score) and with the subjective patient satisfaction (7). For the latter, patients chose one of four answers for following question: “Are you satisfied with the result of the surgery?” (1. very satisfied, 2. satisfied, 3. don’t know, 4. not satisfied).

### Data analysis

Statistical data analysis was performed with SPSS (International Business Machines Corporation, Armonk, NY, USA). With a case number < 30 per group, the data were regarded as not normally distributed. Accordingly, medians and interquartile ranges (IQR) were determined as descriptives. To test for differences between the two surgical groups, *U* tests were performed (VAS, AOFAS, radiographic measurements). For categorized data (ordinal data sets), *U* tests were applied as well (e.g., categorized ROM, patient satisfaction). Alpha level was defined as 0.05.

A sample size calculation was done before study onset on the basis of the AOFAS score. Chan et al. stated a minimal clinically important difference of 19 points on average [21]. Other input parameter of the sample size calculation was a standard deviation of 15 points, an alpha of 0.05, and a beta of 0.1. This resulted in an estimated sample size of 15 per group (30 overall). Sample size calculation was performed with the software G*power 3.1.2 [22]. To account for drop-outs, we chose to include at least 20 patients per group.

## Results

There were 22 cases in the OC group (19 female, 3 male) and 25 in the MIS group (21 female, 4 male). The groups showed no significant differences with regard to the distribution of sexes (*p* = 0.820). Age at time of surgery was 44 (IQR 25) years and 52 (IQR 25) years in the OC and MIS groups, respectively (*p* = 0.117) (Table 1). Baseline clinical data were well comparable between the two groups and are summarized in Tables 2 and 3. Hallux valgus deformity may be accompanied by additional deformity of the proximal phalangeal bone. To address this deformity, a phalangeal akin osteotomy was performed in eight cases in the OC and 13 cases in the MIS group.

**Table 1:** Patient demographics

	OC	MIS
Number of feet	22	25
Right feet	14	13
Left feet	8	12
Bilateral	3	2
Age at time of surgery (years)	44 ± 12	52 ± 7
Female	19	21
Male	3	4

MIS, minimally invasive surgery; OC, open chevron

**Table 2:** Pre-operative clinical data for conventional open and MIS chevron osteotomy

	Conventional distal open chevron (OC)		MIS chevron		
	Md	IQR	Md	IQR	*p* value
VAS pre-operative	6	4	5	4	0.829
AOFAS pre-operative	66.5	32	65	18	0.932
IMA pre-operative	15.15	5.3	14	3.8	0.201
HVA pre-operative	28.25	8.3	26.4	12	0.957

ROM, range of motion; MIS, minimally invasive surgery; OC, open chevron

**Table 3:** Pre-operative range of motion data (categorized)

ROM pre-operative	Open chevron	MIS	*p* value
R1 (ROM < 30°)	4.5%	4%	
R2 (ROM 31°-75°)	50%	40%	0.491
R3 (ROM > 75°)	45.5%	56%	

ROM, range of motion; MIS, minimally invasive surgery; OC, open chevron

None of the post-operative test occasions (6 weeks, 12 weeks, 9 months) showed significant differences between the two groups with regard to VAS or AOFAS score (hypothesis 1, Table 4). Similarly, there were no differences between groups in any of the radiographic outcome parameters (hypothesis 2, Table 4). Post-operative HVA and IMA were similar in both groups and showed significant improvement at all times of post-operative evaluation. No significant differences between the two groups with regard to ROM were observed at any of the post-operative test occasions (6 weeks, 12 weeks, 9 months) (hypothesis 3, Table 5). Regarding patient satisfaction, statistically significant differences were found between MIS and OC surgery 12 weeks post-operatively in favour of the MIS cohort (*p* = 0.022). Six weeks and nine months post-operative, there were no such significant differences in patient satisfaction (hypothesis 4, Table 6). In our study sample, only two patients reported poor satisfaction, one in each group.

**Table 4:** Post-operative clinical data for OC and MIS chevron

	OC		MIS		
	Md	IQR	Md	IQR	*p* value
VAS 6 weeks	1	2	1	3	0.956
VAS 12 weeks	1	2	0	2	0.144
VAS 9 months	0	3	1	2	0.744
AOFAS 6 weeks	72	9	77	17	0.157
AOFAS 12 weeks	83.5	14	85	14	0.237
AOFAS 9 months	90	14	85	15	0.943
AOFAS difference pre-12 weeks	13.5	29	22	22	0.285
AOFAS difference pre-9 months	20	29	23	25	0.781
IMA 6 weeks	7.7	3.2	7.3	1.9	0.565
IMA 12 weeks	7.45	3.1	7.7	2.8	0.898
IMA 9 months	5.85	3.7	6.8	3.0	0.502
HVA 6 weeks	9.9	7.9	10.5	8.7	0.983
HVA 12 weeks	9.15	9.7	8.8	8	0.873
HVA 9 months	8.5	8.8	6.9	7.6	0.839

Md, median; IQR, interquartile range; VAS, Visual Analog Scale; AOFAS, American Orthopedic Foot and Ankle Society–Score; IMA, intermetatarsal angle; HVA, hallux valgus angle; MIS, minimally invasive surgery; OC, open chevron

**Table 5:** Post-operative range of motion in comparison between surgical groups

ROM (R1 (< 30°), R2 (31°–75°), R3 (> 75°)		OC	MIS	*p* value
6 weeks	R1	31.8%	8%	0.075
	R2	63.6%	88%	
	R3	4.5%	4%	
12 weeks	R1	9.1%	0%	0.653
	R2	72.7%	84.0%	
	R3	18.2%	16%	
9 months	R1	5%	0%	0.910
	R2	60%	66.7%	
	R3	35%	33.3%	

MIS, minimally invasive surgery; OC, open chevron; ROM, range of motion

**Table 6:** Patient satisfaction in comparison between surgical groups

		OC	MIS	*p* value
Patient satisfaction 6 weeks	Very satisfied	54.5%	80%	0.061
	Satisfied	22.7%	12%	
	Don’t know	13.6%	4%	
	Not satisfied	4.5%	4%	
Patient satisfaction 12 weeks	Very satisfied	63.6%	92%	0.022*
	Satisfied	13.6%	4%	
	Don’t know	18.2%	0%	
	Not satisfied	4.5%	4%	
Patient satisfaction 9 months	Very satisfied	70%	62.5%	0.736
	Satisfied	15%	25%	
	Don’t know	0%	4.2%	
	Not satisfied	15%	8.3%	

MIS, minimally invasive surgery; OC, open chevron

Besides the hypotheses-based analyses, the following findings are reported: Soft tissue irritation caused by the K wire, which had to be removed in 12 feet. In contrast, in the OC cohort, the screw had to be removed in two feet (2 out of 22). Hardware removal did not affect satisfaction ratings. All cases (12 out of 25 feet) with K wire irritation led to local pain and made K wire removal necessary. In the OC group, hard ware had to be removed in one case due to local pain and soft tissue irritation as well. In the second case, removal was mandatory due to patient wish. In consideration of bone healing, we would not recommend removal of hard ware before week 12 after surgery. Moreover, removal in asymptomatic cases without soft tissue irritation screw or K wire removal is not necessary. There were no cases of fractures, loss of fixation, or avascular necrosis. One case of a hallux varus was identified in the OC group; it remained subclinical and did not need revision surgery. Three feet in the OC and one in the MIS group presented radiologically a mild recurrence of deformity without the need for revision so far. Clinically, all these patients reported a “good” outcome in terms of VAS, AOFAS, and outcome score.

## Discussion

It is regarded as the most important finding of this study, that the results of the minimally invasive chevron technique were comparable with those of the classical open technique. We identified no difference between groups with regard to (1) clinical outcome score (VAS, AOFAS) (hypothesis 1 rejected), (2) radiographic outcome parameters (HVA, IMA) (hypothesis 2 rejected), or (3) range of motion of the first metatarsophalangeal joint (hypothesis 3 rejected). However, patient satisfaction was significantly superior in the MIS group 12 weeks post-operatively, but not at six weeks or nine months (hypothesis 4 accepted). Both groups experienced a significant pre-to-post-operative correction in terms of the IMA. Over the first nine post-operative months, no significant loss of correction was detected, neither in the minimally invasive nor in the open group.

In an attempt to compare our findings with the reports made by previous researchers, we found 14 retrospective studies outlining results of minimally invasive hallux valgus surgery. Most of them presented good clinical outcome in mid-term [12, 23] as well as in long-term follow-up after ten years [24]. Radiological outcome also revealed good results [9, 12]. However, no prospective randomized studies comparing a minimally invasive and an open chevron technique have been published so far. This problem was also issued by Trnka et al. who could not make a definite recommendation in their systematic review analysis [25], since the majority of the studies appeared to be level IV studies performed at centers offering primarily minimally invasive hallux valgus surgery and prospective randomized studies are lacking to date. What is more, the majority of the published studies presented outcome after linear distal metatarsal osteotomies [14], which might have an impact on the joint line. Iyer et al. found higher recurrence rates in individuals with pathological joint lines, whereas another study demonstrated a probability for a metatarsal misload after Reverdin-Isham procedures [26, 27]. Other authors showed already good clinical and radiological results with a minimally invasive chevron technique, but our analysis is the first to compare a minimally invasive chevron type and an open chevron osteotomy in a randomized manner [9, 10, 12]. We consider the chevron type superior to linear osteotomy techniques, since maintenance of the joint line and physiological positioning of the metatarsal head in the coronal plain are more likely with this technique.

Average IMA correction was a marker to determine the corrective potential of a metatarsal osteotomy. Complete reduction of the IMA should result in complete repositioning of the sesamoids as well. Recent literature reports a correlation between IMA and sesamoid position in hallux deformity as well as after hallux valgus correction [28, 29]. We believe that the IMA expresses whether the shift of the metatarsal head is sufficient. In our study, we found good correction of the IMA in both groups (Table 4), comparable to published results in the literature [8, 23, 24]. Like the IMA, analysis of the HVA also showed a comparable correction with negligible loss of correction in both groups. A phalangeal osteotomy as well as the quality of the lateral release have a direct impact, the metatarsal osteotomy an indirect impact on this angle. Correction of the HVA in our study (compare Table 2 and Table 4) is in good accordance with the published results in the literature [12].

It is well known that after hallux surgery, clinical scores show significant improvement [30, 31]. In retrospective studies of MIS hallux valgus procedures, this finding was also detected [23, 24]. Our data support this finding as well. With the AOFAS forefoot score, we detected a significant improvement in both cohorts without differences between the two groups (Table 4). This was observed after six and 12 weeks as well as at follow-up after nine months post-operative. A second functional outcome parameter was determined by analyzing the ROM of the greater toe joint. In young athletes, maintenance of the pre-operative levels was recently described as a predictive marker for excellent outcome [32]. Our data show similar ROM values in both cohorts at all points of follow-up with maintenance of the pre-operative ROM (Table 5).

Evaluation of VAS showed significant improvement at all time points in both groups to a comparable extent (Table 4). Residual pain six months after open hallux surgery with improvement over the following 18 months has been reported [33]. In our study, we could not detect a significant difference between the two groups at any of the time points of our survey, although minor soft tissue irritation was expected with the minimally invasive technique. In contrast to comparable pain levels, we found a statistical difference in patient satisfaction after three months in favor of the MIS cohort, but not at the other time points. This finding may be attributed to minor soft tissue irritation from this technique. Individuals were assigned to the two groups by lot. However, age at time of surgery was higher in the OC group. Since we found no differences in radiological outcome and comparable clinical results between the two groups, we think that this finding can be left out of consideration.

**Fig. 5:**
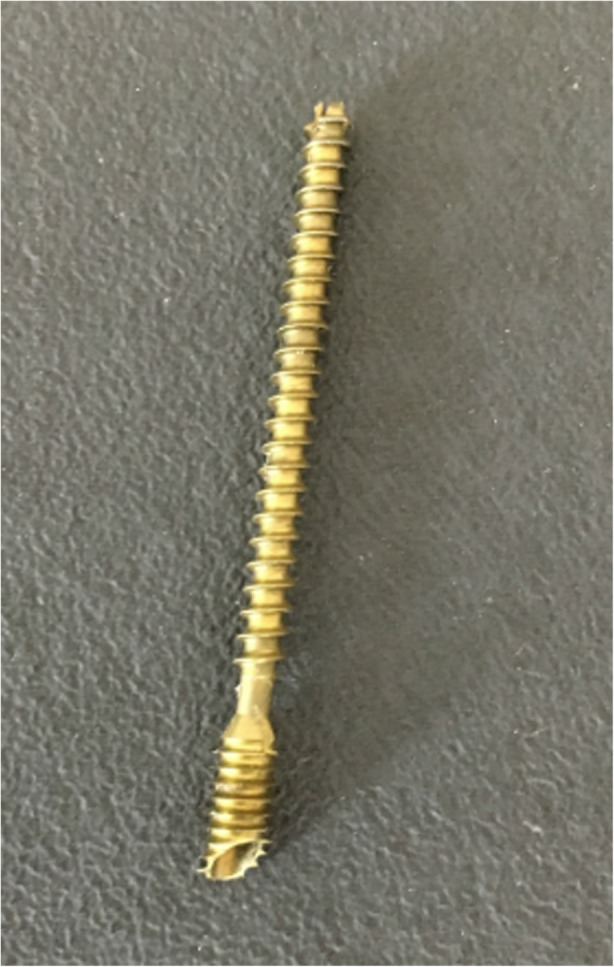
Cannulated oblique headed 3.0 mm compression screw

Another remarkable soft tissue irritation event was determined with our investigation. K wire fixation of the metatarsal head in the MIS group led to an extraordinarily high need for hardware removal (12 out of 25 feet). In contrast, in the OC group, only two Fusion and Reconstruction System (FRS) screws had to be removed, which was credited to the oblique insertion of the plain headed screw in one case and to a patient wish in the other case. As a result of this finding, we have meanwhile changed the fixation method in MIS chevron osteotomy. Since we commenced implementation of a cannulated oblique headed compression screw (3.0 mm—MICA-Chevron-Screw, Wright-Medical), no further hardware removal has been necessary (Fig. 5). Therefore, we cannot recommend K wires for routine MIS cases, whereas the use of the oblique headed compression screw seems to result in the lowest rate of soft tissue irritation.

The following limitations are acknowledged. First, the investigation was performed at one single centre and is based on cases performed by a single senior surgeon with specialization in foot and ankle surgery (GK). Second, our follow-up of nine months is regarded as a preliminary result. The two cohorts will continue to be followed.

The strength of the study is that it is to our best knowledge the first study to apply a randomized controlled prospective design. Therefore, this study is regarded as relevantly contributing new scientific knowledge.

The findings are also regarded as clinically relevant. This is because there is an increasing demand for minimally invasive surgical techniques in hallux valgus surgery. This demand is driven by patients and by surgeons attempting to meet the patients’ needs. This is also seen in the increasing number of publications dealing with minimally invasive hallux valgus surgery. However, until now, no study has been published with a prospective randomized design.

From our findings, we conclude that the outcome of MIS chevron osteotomy is comparable to that of the conventional open technique. We could not identify a difference with regard to clinical outcome score (VAS, AOFAS), radiographic outcome parameters, or range of motion. However, patient satisfaction was significantly superior in the MIS group 12 weeks post-operative.

## References

[1] Austin DW, Leventen EO (1981) A new osteotomy for hallux valgus: a horizontally directed “V” displacement osteotomy of the metatarsal head for hallux valgus and primus varus. Clin Orthop Relat Res (157):25–30 7249456

[2] Trnka HJ, Zembsch A, Easley ME, Salzer M, Ritschl P, Myerson MS (2000). The chevron osteotomy for correction of hallux valgus. Comparison of findings after two and five years of follow-up. J Bone Joint Surg Am.

[3] Oh IS, Choi SW, Kim MK, Lee SY, Lee JS (2008) Clinical and radiological results after modified distal metatarsal osteotomy for hallux valgus. Foot Ankle Int 29(5):473–477. 10.3113/FAI.2008.0473 10.3113/FAI-2008-0473 10.3113/FAI-2008-0473 18510898

[4] Choi WJ, Yoon HK, Yoon HS, Kim BS, Lee JW (2009). Comparison of the proximal chevron and Ludloff osteotomies for the correction of hallux valgus. Foot Ankle Int.

[5] Thordarson D, Ebramzadeh E, Moorthy M, Lee J, Rudicel S (2005). Correlation of hallux valgus surgical outcome with AOFAS forefoot score and radiological parameters. Foot Ankle Int.

[6] Park CH, Jang JH, Lee SH, Lee WC (2013). A comparison of proximal and distal chevron osteotomy for the correction of moderate hallux valgus deformity. Bone Joint J.

[7] Vopat BG, Lareau CR, Johnson J, Reinert SE, DiGiovanni CW (2013). Comparative study of scarf and extended chevron osteotomies for correction of hallux valgus. Foot Ankle Spec.

[8] Brogan K, Voller T, Gee C, Borbely T, Palmer S (2014). Third-generation minimally invasive correction of hallux valgus: technique and early outcomes. Int Orthop.

[9] Jowett CRJ, Bedi HS (2017). Preliminary results and learning curve of the minimally invasive chevron akin operation for hallux valgus. J Foot Ankle Surg.

[10] Lee M, Walsh J, Smith MM, Ling J, Wines A, Lam P (2017). Hallux valgus correction comparing percutaneous chevron/akin (PECA) and open scarf/akin osteotomies. Foot Ankle Int.

[11] Redfern D, Perera AM (2014). Minimally invasive osteotomies. Foot Ankle Clin.

[12] Brogan K, Lindisfarne E, Akehurst H, Farook U, Shrier W, Palmer S (2016). Minimally invasive and open distal chevron osteotomy for mild to moderate hallux valgus. Foot Ankle Int.

[13] Crespo Romero E, Penuela Candel R, Gomez Gomez S, Arias Arias A, Arcas Ordono A, Galvez Gonzalez J, Crespo Romero R (2017). Percutaneous forefoot surgery for treatment of hallux valgus deformity: an intermediate prospective study. Musculoskelet Surg.

[14] Maffulli N, Longo UG, Marinozzi A, Denaro V (2011). Hallux valgus: effectiveness and safety of minimally invasive surgery. A systematic review. Br Med Bull.

[15] Hardy RH, Clapham JC (1951). Observations on hallux valgus; based on a controlled series. J Bone Joint Surg Br.

[16] Venning P (1951). Sources of error in the production and measurement of standard radiographs of the foot. Br J Radiol.

[17] Wulker N (1997). Hallux valgus. Orthopade.

[18] Smith RW, Reynolds JC, Stewart MJ (1984). Hallux valgus assessment: report of research committee of American Orthopaedic Foot and Ankle Society. Foot Ankle.

[19] Miller JW (1974). Distal first metatarsal displacement osteotomy. Its place in the schema of bunion surgery. J Bone Joint Surg Am.

[20] Mitchell CL, Fleming JL, Allen R, Glenney C, Sanford GA (1958). Osteotomy-bunionectomy for hallux valgus. J Bone Joint Surg Am.

[21] Chan HY, Chen JY, Zainul-Abidin S, Ying H, Koo K, Rikhraj IS (2017). Minimal clinically important differences for American Orthopaedic Foot & Ankle Society Score in hallux valgus surgery. Foot Ankle Int.

[22] Faul F, Erdfelder E, Lang AG, Buchner A (2007). G*power 3: a flexible statistical power analysis program for the social, behavioral, and biomedical sciences. Behav Res Methods.

[23] Giannini S, Faldini C, Nanni M, Di Martino A, Luciani D, Vannini F (2013). A minimally invasive technique for surgical treatment of hallux valgus: simple, effective, rapid, inexpensive (SERI). Int Orthop.

[24] Faour-Martin O, Martin-Ferrero MA, Valverde Garcia JA, Vega-Castrillo A, de la Red-Gallego MA (2013). Long-term results of the retrocapital metatarsal percutaneous osteotomy for hallux valgus. Int Orthop.

[25] Trnka HJ, Krenn S, Schuh R (2013). Minimally invasive hallux valgus surgery: a critical review of the evidence. Int Orthop.

[26] Iyer S, Demetracopoulos CA, Sofka CM, Ellis SJ (2015). High rate of recurrence following proximal medial opening wedge osteotomy for correction of moderate hallux valgus. Foot Ankle Int.

[27] Rodriguez-Reyes G, Lopez-Gavito E, Perez-Sanpablo AI, Galvan Duque-Gastelum C, Alvarez-Camacho M, Mendoza-Cruz F, Parra-Tellez P, Vazquez-Escamilla J, Quinones-Uriostegui I (2014). Dynamic plantar pressure distribution after percutaneous hallux valgus correction using the Reverdin-Isham osteotomy. Rev Invest Clin 66 Suppl.

[28] Agrawal Y, Desai A, Mehta J (2011). Lateral sesamoid position in hallux valgus: correlation with the conventional radiological assessment. Foot Ankle Surg.

[29] Mahadevan D, Lines S, Hepple S, Winson I, Harries W (2016). Extended plantar limb (modified) chevron osteotomy versus scarf osteotomy for hallux valgus correction: a randomised controlled trial. Foot Ankle Surg.

[30] Saro C, Jensen I, Lindgren U, Fellander-Tsai L (2007). Quality-of-life outcome after hallux valgus surgery. Qual Life Res.

[31] Jeuken RM, Schotanus MG, Kort NP, Deenik A, Jong B, Hendrickx RP (2016). Long-term follow-up of a randomized controlled trial comparing scarf to chevron osteotomy in hallux valgus correction. Foot Ankle Int.

[32] Giotis D, Paschos NK, Zampeli F, Giannoulis D, Gantsos A, Mantellos G (2016). Modified Chevron osteotomy for hallux valgus deformity in female athletes. A 2-year follow-up study. Foot Ankle Surg.

[33] Chen JY, Ang BF, Jiang L, Yeo NE, Koo K, Singh Rikhraj I (2016). Pain resolution after hallux valgus surgery. Foot Ankle Int.

